# Ultrasonography for suspected mild-to-moderate acute left colonic diverticulitis: a prospective head-to-head study with computed tomography

**DOI:** 10.3389/fradi.2026.1829813

**Published:** 2026-05-28

**Authors:** Nuria Roson, Andreu Antolin, Miriam Flores-Yelamos, M. Victoria Garriga, Xavier Pruna, Salvador Pedraza, Manuel Lopez-Cano, Josep M. Badia

**Affiliations:** 1 Department of Radiology, Hospital Universitari Vall d’Hebron, Barcelona, Spain; 2 Department of General and Digestive Surgery, Hospital General de Granollers, Granollers, Spain; 3 Universitat Internacional de Catalunya, Barcelona, Spain; 4 Department of Radiology, Institut de Diagnòstic per la Imatge (IDI), Hospital Universitari Josep Trueta, Girona, Spain; 5 Department of Radiology, Hospital General de Granollers, Granollers, Spain; 6 Department of Radiology, Hospital Clínic de Barcelona, Barcelona, Spain; 7 Department of General and Digestive Surgery, Hospital Universitari Vall d’Hebrón, Barcelona, Spain

**Keywords:** acute colonic diverticulitis, computed tomography, prospective studies, sensitivity and specificity, ultrasonography

## Abstract

**Background:**

The preferred imaging modality for acute left colonic diverticulitis (ALCD) remains controversial. This prospective study compares ultrasonography (US) and computed tomography (CT) in selected patients with clinically suspected mild-to-moderate localised ALCD.

**Materials and methods:**

Consecutive patients presenting to the emergency department with suspected localised mild-to-moderate ALCD underwent both US and CT within 1 h and before treatment initiation. US and CT examinations were performed and interpreted by three trained abdominal radiologists. A multidisciplinary consensus panel established the reference (“final”) diagnosis, against which both tests were compared. Sensitivity, specificity, positive predictive value (PPV), and negative predictive value (NPV) were calculated for each modality. Agreement between US and CT for severity stratification was also assessed. This study was not designed as a non-inferiority or equivalence analysis.

**Results:**

Sixty-three patients were included in the study; 50 (79.4%) had a final diagnosis of ALCD. US correctly identified 47/50 patients with ALCD, yielding a sensitivity of 97.9%, specificity of 91.7%, PPV of 97.9%, and NPV of 91.7%, while CT showed a sensitivity of 98.0%, specificity of 92.3%, PPV of 98.0%, and NPV of 92.3%. A moderate agreement was found between the two treatment modalities for differentiating stage 0 (mild-to-moderate) from stage >0 (severe) localised ALCD (*κ* = 0.568, *p* < 0.001), which increased when US was performed by a more experienced operator (*κ* = 0.70, *p* < 0.001). Higher C-reactive protein levels were associated with higher radiological severity (*p* = 0.014).

**Conclusion:**

In this selected prospective cohort, ultrasonography performed by experienced abdominal radiologists showed diagnostic estimates similar to CT and identified most ALCD cases. Given the limited sample size, operator dependence, and non-independent reference standard, these findings should be interpreted cautiously and are most applicable to specialised centres with dedicated ultrasound expertise.

**Clinical Trial Registration**: ClinicalTrials.gov, NCT05323968.

## Introduction

Acute colonic diverticulitis is a common cause of urgent medical attendance (1). In Western countries, diverticula most often involve the sigmoid and descending colon. Although fewer than 5% of individuals with diverticulosis are estimated to develop acute left colonic diverticulitis (ALCD), diverticulosis affects 50%–60% of people older than 60 years, resulting in a substantial disease burden (2, 3). Diverticulosis has also increased among younger patients in recent years despite its association with age (4, 5). Because ALCD symptoms are often non-specific and overlap with other acute abdominal conditions, imaging is required to exclude alternative causes, confirm the diagnosis, and assess extent and complications to guide management (1, 6). Computed tomography (CT) and abdominal ultrasonography (US) are the most commonly used modalities, yet their preferred use remains controversial across guidelines (7). European guidelines generally recommend US as the first-line test, whereas North American guidance favours CT as the initial and most effective modality.

This study tested the hypothesis that a properly performed US examination by trained radiologists provides sufficient accuracy for reliable diagnosis and assessment of ALCD. The diagnostic accuracy of US in patients with clinically suspected ALCD was evaluated and compared with CT, using a consensus “final diagnosis” from three experts as the reference standard.

## Methods

### Study design

A prospective longitudinal cohort study was conducted to assess the diagnostic accuracy of US and CT. The study was conducted under expert daytime conditions in a teaching-hospital radiology department with extensive experience in emergency abdominal ultrasonography. Sixty-three consecutive patients presenting to the Emergency Department with a clinical suspicion of ALCD were included in the study. Body mass index was not an exclusion criterion. Patients presenting outside regular working hours (08:00–17:00 on weekdays), during holidays, or on night shifts were not included in order to ensure image acquisition and interpretation by the same group of abdominal radiologists.

Patients with suspected localised, mild-to-moderate ALCD underwent US followed by CT. The interval between examinations was <1 h in all patients, and both techniques were performed before initiation of treatment. US and CT were performed, interpreted, and reported by three consultant radiologists from the Abdominal Radiology Unit (Radiologists A–C) with experience in abdominal imaging; Radiologists A and B had >15 years' experience and Radiologist C >5 years. For each patient, two radiologists were assigned according to availability: one performed US and the other CT, blinded to the counterpart examination and aware only of the clinical indication. Blood test results were not available to the radiologists at the time of imaging. Awareness of the suspected diagnosis reflected routine clinical practice but may have introduced diagnostic expectation bias.

### Definitions and variables

**Figure 1:**
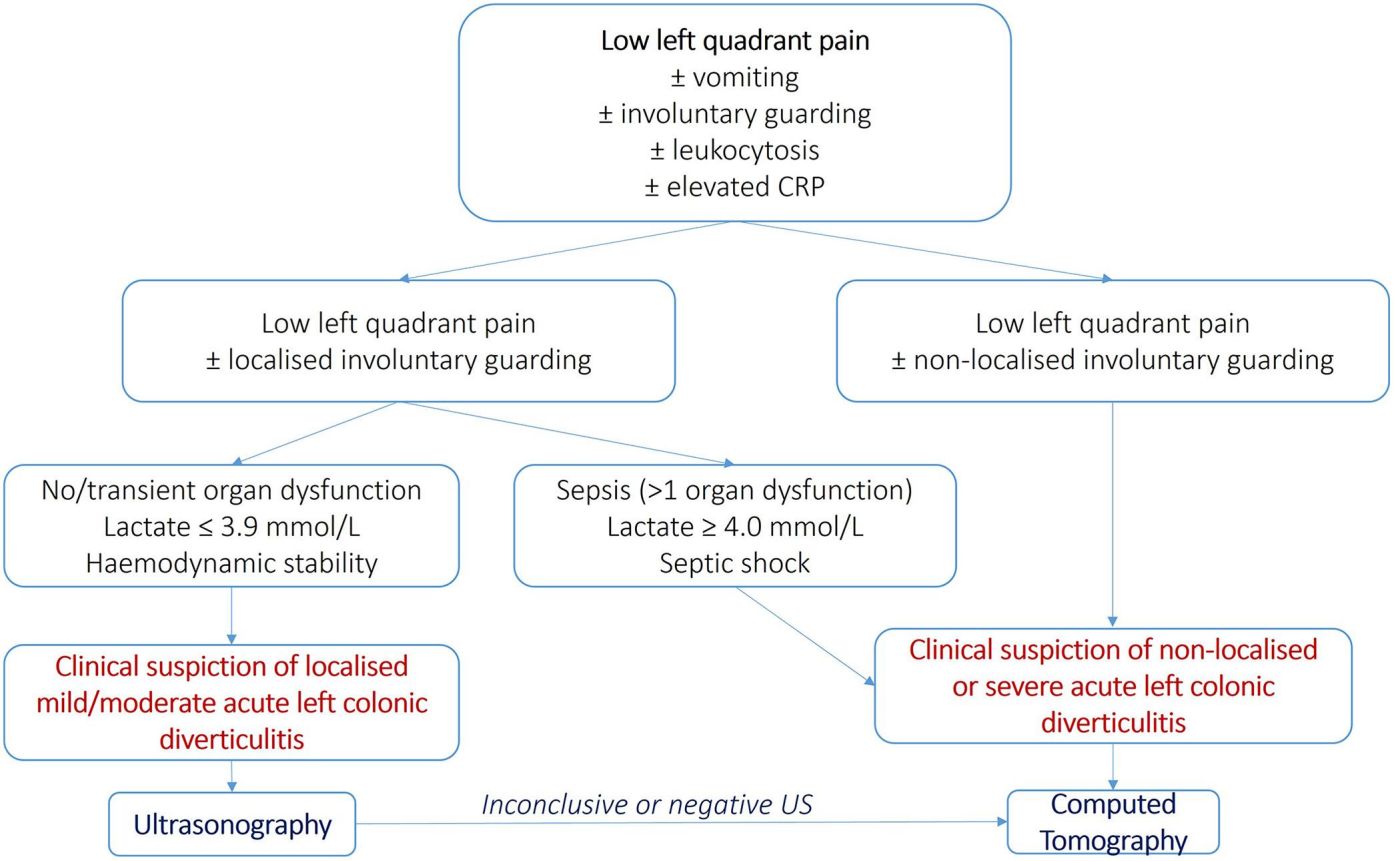
Standard institutional protocol for ordering imaging tests in patients with suspected acute left colonic diverticulitis. In the present study, both imaging tests were performed in all included patients.

Clinical suspicion of ALCD was defined as left lower quadrant abdominal pain, with or without a palpable mass, tenderness, or involuntary guarding. Secondary symptoms considered in the diagnostic algorithm were fever, nausea, vomiting, and diarrhoea (8, 9). According to the institutional pathway, localised mild-to-moderate ALCD was defined by left lower quadrant pain, absence of peritonitis in other abdominal quadrants, and absence of sepsis or organ failure. The standard-of-care imaging pathway is shown in Figure 1. Both imaging tests were performed in all patients.

Demographic, clinical, and laboratory data were collected prospectively from electronic medical records. Additional variables included treatments, complications, need for further imaging, surgical procedures, and length of stay. Radiologist performance for US diagnosis of acute diverticulitis was also recorded.

ALCD severity was graded using the modified Neff classification based on CT findings (10). US criteria were adapted from the CT-based classification to apply identical radiological criteria to both modalities. Minimum findings for stage 0 were diverticula, mural thickening, and pericolic fat inflammation. Mural thickening was defined as a colonic wall thickness ≥4 mm on US. Stage Ia comprised localised pneumoperitoneum (air bubbles adjacent to the colonic wall) and stage Ib an abscess <4 cm. Stage 0 was considered uncomplicated diverticulitis, whereas stages Ia and Ib were considered locally complicated. Complicated diverticulitis included pelvic abscess >4 cm (stage II), intra-abdominal abscess outside the sigmoid region and pelvis (stage III), and profuse pneumoperitoneum with or without free intraperitoneal fluid (stage IV).

The reference standard (“final diagnosis”) was established by a three-member panel (two surgeons, one radiologist) not involved in prior image assessment. Diagnosis was reached by consensus using pre-admission history; laboratory, microbiological, US, and CT findings; response to medical therapy; surgical findings when available; discharge reports; post-discharge investigations (e.g., colonoscopy); and clinical and radiological follow-up over 2 years after the index admission. Severity class was determined by imaging alone and was not incorporated into the reference diagnosis. The consensus panel did not participate in the original acquisition or primary reporting of the imaging examinations. However, because both US and CT findings were available to the panel during final diagnosis adjudication, the reference standard was not fully independent from the index tests.

### Ultrasonography

US was performed with the patient supine using both low-frequency convex and high-frequency linear transducers. Scanning started in the left iliac fossa, with the area of maximal tenderness (indicated by the patient) used as the focal point. The sigmoid colon was targeted in the region between the left anterior superior iliac spine and the bladder, using the midpoint as the landmark for initial identification. After identification in the left iliac fossa or suprapubic region, the sigmoid colon was followed in transverse and longitudinal planes to its junction with the descending colon, which was then assessed with sweeping transverse and longitudinal scans. When an adequate acoustic window was available, a high-frequency linear transducer was subsequently used for detailed assessment of bowel-wall thickening, inflamed diverticula, and superficial pericolic complications.

Mural oedema was defined as a thickened, hypoechoic colonic wall and/or inflamed diverticula. Vascularity and hypervascularity of the bowel wall and pericolic fat were assessed with colour Doppler to support identification of inflammatory changes, including low-PRF settings and superb microvascular imaging (SMI) for slow-flow detection. In all cases, the examination included all four abdominal quadrants and was not limited to the left lower quadrant. Colour Doppler assessment was recommended as an adjunct to greyscale examination when technically feasible, but its use was not mandatory in every case and remained operator-dependent. For this reason, Doppler findings were considered supportive rather than required diagnostic criteria.

### Technical characteristics

US scans were performed with an Aplio 500 (Canon, Tokyo, Japan) using a 1–6 MHz convex transducer and a 5–14 MHz linear transducer. All CT scans were performed using a Somatom Emotion 6-slice model (Siemens, Erlangen, Germany). The slice thickness used was 3.0 mm with a 1.5 pitch. The studies were performed after administration of intravenous contrast (Ultravist300 mg/mL; Bayer Hispania, S.L., Sant Joan Despí, Spain).

### Ethical issues

The study was approved by the hospital's Clinical Research Ethics Committee with code CEIC20173001, and in all cases, informed consent was obtained for additional CT or US examinations. Anonymity and data confidentiality (access to records, data encryption, and data storage) were guaranteed throughout the research process. Confidential patient information was protected in accordance with the European General Data Protection Regulation (GDPR). The project was registered at ClinicalTrials.gov Identifier: NCT05323968. The manuscript conforms to the Standards for the Reporting of Studies of Diagnostic Accuracy (STARD) (11).

### Statistical analysis

Qualitative variables were summarised as frequencies and percentages. Quantitative variables were summarised using mean and standard deviation, or median with quartiles, minimum, and maximum. The qualitative variables were compared between complicated and uncomplicated ALCD using the *χ*² test or Fisher's exact test, as appropriate. The quantitative variables were compared between two independent groups using Student's *t*-test for normally distributed data and the Mann–Whitney *U* test for non-normal data.

Diagnostic accuracy of US and CT for mild-to-moderate ALCD was defined by agreement with the expert-panel “final diagnosis” and expressed as sensitivity, specificity, positive predictive value, and negative predictive value. For the primary analysis, indeterminate US examinations were excluded from the 2 × 2 diagnostic accuracy table. Agreement between US and CT for classifying uncomplicated (stage 0) vs. complicated (stage >0) ALCD was assessed using Cohen's kappa, including agreement for individual modified Neff features (pericolic air bubbles, pericolic fluid, abscess, free fluid, and pneumoperitoneum). Statistical significance was set at *p* < 0.05. Analyses were performed using R (R-Gui v4; The R Foundation for Statistical Computing, Vienna, Austria).

## Results

**Figure 2:**
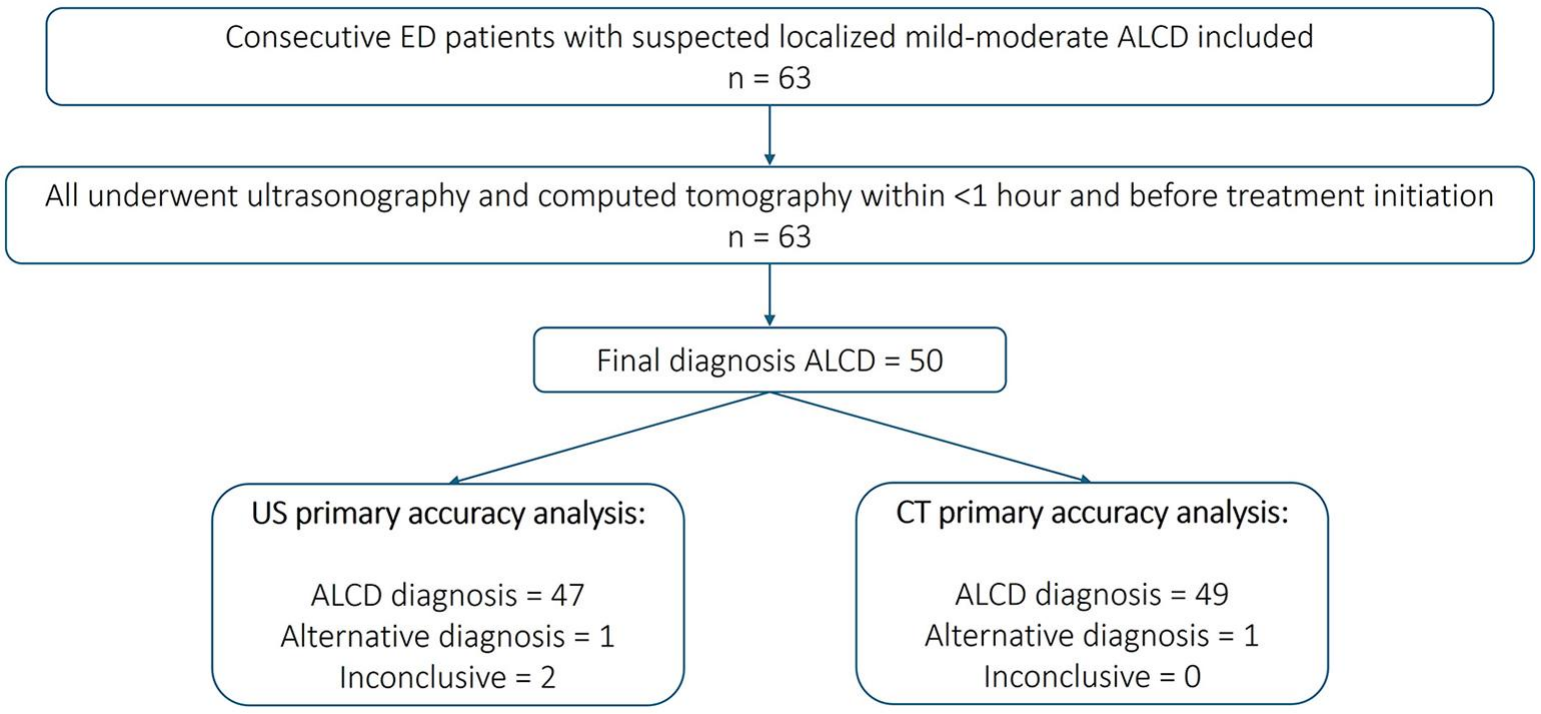
STARD flow diagram of participant inclusion, imaging assessment, and final diagnostic classification.

Over 17 months, 63 patients with clinically suspected ALCD were included in the study, of whom 50 (79.4%) were finally diagnosed with ALCD (Figure 2). The remaining 13 (20.6%) had another cause of acute abdominal pain (Supplementary Table S1). Among patients with a final diagnosis of ALCD, the mean age was 55.1 ± 11.9 years (range 39–82 years) for men and 66.2 ± 13.4 years (range 46–88 years) for women (*p* = 0.003). The clinical and laboratory data of these patients are presented in Table 1.

**Table 1:** Clinical symptoms and analytical parameters of the patients included in the series.

Clinical and laboratory data	Whole series *N* = 63, *n* (%)	ALCD *N* = 50, *n* (%)	Other diagnostics *N* = 13, *n* (%)
Pain in the lower left quadrant	63 (100)	50 (100)	13 (100)
Involuntary guarding/rebound tenderness	37 (58.7)	30 (60)	5 (38.5)
Palpable mass	2 (3.2)	2 (4)	0
Nausea	17 (27.0)	12 (24)	5 (38.5)
Vomiting	11 (17.4)	10 (20)	1 (7.7)
Diarrhoea	7 (11.1)	4 (8)	3 (23.2)
Rectal bleeding	3 (4.7)	3 (6)	0
Constipation	3 (4.8)	3 (6)	0
Fever (>38°)	5 (8.0)	5 (10)	0
White blood cell count >11,000/ cells/µL	39 (61.9)	31 (62)	8 (61.5)
White blood cell count <4,000/cells/µL	1 (1.5)	1 (2)	0
C-reactive protein >0,1 mg/dL	63 (100)	50 (100)	13 (100)
SIRS criteria	6 (9.5)	6 (12)	0

In the US assessment, 47 patients received a diagnosis of ALCD, 13 were assigned an alternative diagnosis, and three examinations were considered indeterminate. Among the 50 patients with a final diagnosis of ALCD, US correctly identified 47 patients; one patient with ALCD was classified as not having diverticulitis and two additional patients with ALCD had indeterminate US findings. No patient was excluded because of a bowel-gas artefact or an elevated body mass index.

CT classified 49 patients as having ALCD and 14 as having an alternative diagnosis, with no indeterminate CT examinations. Among the 50 patients with a final diagnosis of ALCD, CT correctly identified 49 patients; the remaining patient was classified as having diverticulosis without diverticulitis. Among patients with a final diagnosis other than ALCD, one patient was classified as having ALCD by both imaging techniques but was ultimately adjudicated as having acute gastroenteritis.

The diagnostic classification of US and CT according to final diagnosis is presented in Table 2. Overall, US achieved a sensitivity of 97.9%, specificity of 91.7%, positive predictive value (PPV) of 97.9%, and negative predictive value (NPV) of 91.7%, whereas CT achieved a sensitivity of 98.0%, specificity of 92.3%, PPV of 98.0%, and NPV of 92.3% (Table 3).

**Table 2:** Simplified diagnostic classification of ultrasonography (US) and computed tomography (CT) according to final diagnosis.

Ultrasonography			
Final diagnosis	US: ALCD	US: non-ALCD/other diagnosis	US: indeterminate
ALCD (50)	47	1^a^	2
Non-ALCD (13)	1	11^b^	1

**Computed tomography**			
**Final diagnosis**	**CT: ALCD**	**CT: non-** **ALCD/other** **diagnosis**	
ALCD (50)	49	1	
Non-ALCD (13)	1	12	

a Final diagnosis of ALCD but classified on US as diverticulosis without diverticulitis.

b Includes 1 indeterminate US examination in a patient with a final diagnosis other than ALCD; indeterminate US examinations were excluded from the primary diagnostic accuracy analysis.

**Table 3:** Comparison of the sensitivity, specificity, positive predictive value, and negative predictive value with confidence intervals between ultrasound (US) and computed tomography (CT) for the final diagnosis of patients with suspected acute left colonic diverticulitis (ALCD).

Diagnostic accuracy parameter	US	CT
Sensitivity (%, 95% CI)	97.9 (87.5; 99.9)	98.0 (88.0; 99.9)
Specificity (%, 95% CI)	91.7 (59.8; 99.6)	92.3 (62.1; 99.6)
PPV (%, 95% CI)	97.9 (87.5; 99.9)	98.0 (88.0; 99.9)
NPV (%, 95% CI)	91.7 (59.8; 99.6)	92.3 (62.1; 99.6)

PPV, positive predictive value; NPV, negative predictive value.

Among the 47 patients with a final diagnosis of ALCD, US classified 34 (72.3%) patients as stage 0 and 13 (27.7%) patients as higher stage. For CT, 30 (63.8%) and 19 (36.2%) patients were classified as stage 0 and >0, respectively. These results do not include the patient in whom both techniques made a diagnosis of ALCD (stage 0), but the final diagnosis was acute gastroenteritis (Table 4).

**Table 4:** Correlation of acute left colonic diverticulitis stages according to the modified Neff classification on computed tomography (CT) and ultrasound (US).

Classification	US (*n* = 47), *n* (%)	CT (*n* = 49) , *n* (%)
Modified Neff 0	34 (72.3)	30 (61.2)
Modified Neff Ia	8 (17.0)	12 (24.5)
Modified Neff Ib	3 (6.4)	4 (8.2)
Modified Neff II	2 (4.3)	2 (4.1)
Modified Neff III	0 (0)	0 (0)
Modified Neff IV	0 (0)	1 (2.0)

A moderate level of agreement was detected between both techniques for differentiating the presence of uncomplicated (stage 0) or complicated (stage >0) ALCD with a Kappa of 0.568 (0.315, 0.821, *p* < 0.001). However, the individual performance of each of the three radiologists was different. It was observed that the agreement between the two techniques was substantial in Radiologist A (Kappa 0.700; 0.429, 0.971, *p* < 0.001) and weakened (Kappa 0.262; 0.287, 0.812, *p* = 0.194) in the other two. This trend was also observed when assessing the classification stage (Table 5), in which Radiologist A had better agreement between US and CT than the other two radiologists with less experience when staging ALCD (stage 0: 69% vs. 78.9%; stage >0: 31% vs. 21.1%).

**Table 5:** Comparison of the results obtained according to the modified Neff classification between the abdominal radiologist with the most experience in ultrasound in the setting of acute abdominal pain and the two remaining radiologists participating in the study.

Classification	Neff-modified 0, *n* (%)	Neff-modified Ia, *n* (%)	Neff-modified Ib, *n* (%)	Neff-modified II, *n* (%)
Radiologist A	20 (69.0)	5 (17.2)	2 (6.9)	2 (6.9)
Radiologists B–C	15 (78.9)	3 (15.8)	1 (5.3)	0 (0)

**Figure 3:**
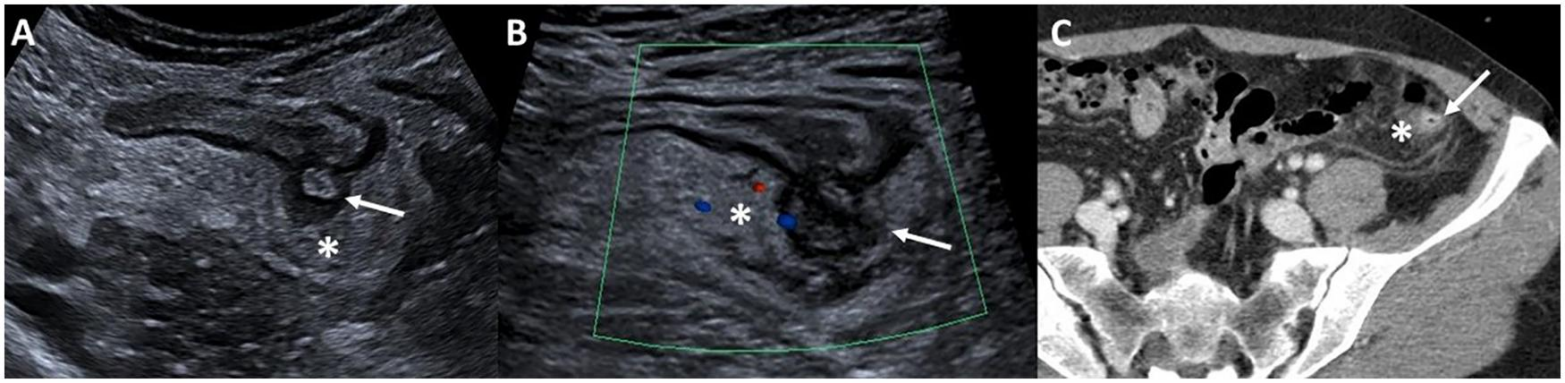
Acute left colonic diverticulitis stage 0. US images obtained with convex transducer **(A)** and lineal transducer **(B)** in the axial plane show a mural thickening diverticulum (arrow) with echogenic fat tissue around it (*). A contrast-enhanced CT scan of the same segment **(C)** shows hyperattenuating mural diverticulum (arrow) surrounded by pericolic inflammation (*).

**Figure 4:**
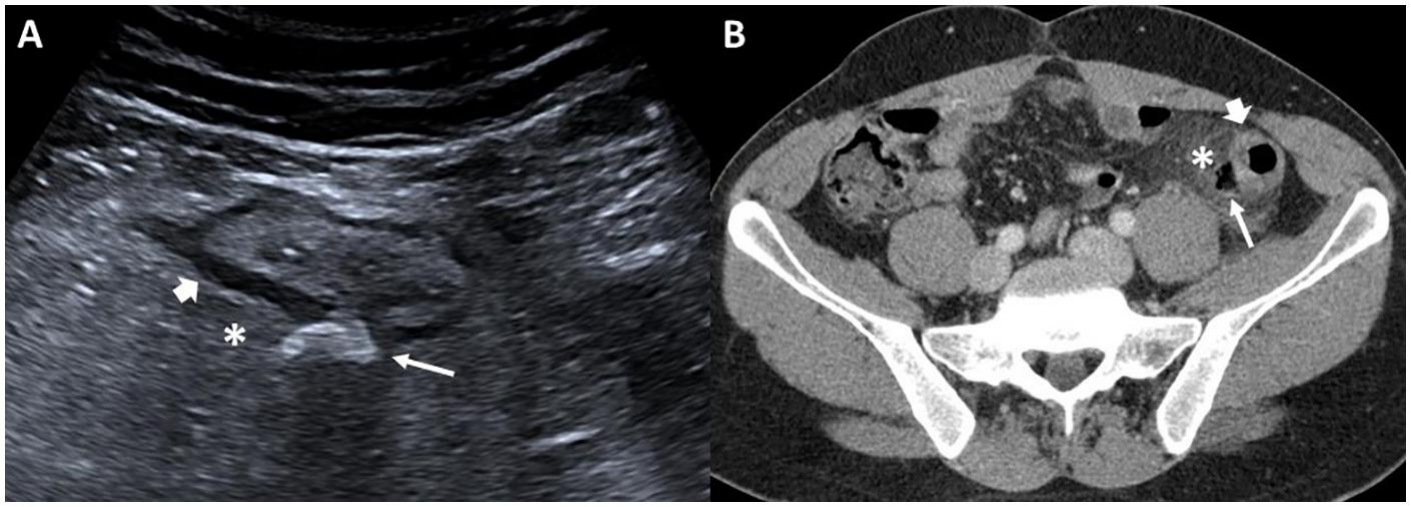
Acute left colonic diverticulitis stage 1a. A US image obtained with a convex transducer in an oblique plane **(A)** shows a thickening of the left colon (short arrow), with hyperechoic fat (*) with echogenic foci adjacent to the wall of the colon (large arrow). A contrast-enhanced CT in the axial plane **(B)** demonstrates similar findings: thickening of the left colonic wall (short arrow) with pericolic inflammation (*) and a bubble of gas (short arrow).

**Figure 5:**
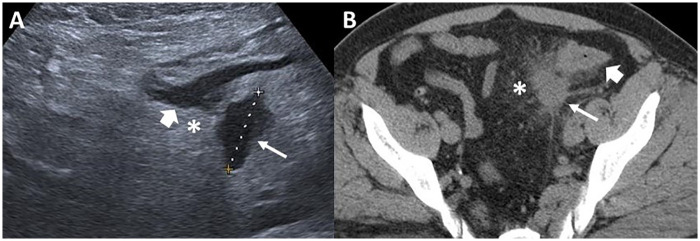
Acute left colonic diverticulitis stage 1b. A US image obtained with a convex transducer **(A)** shows a thickening of the wall of the colon (short arrow), hyperechogenic fat, and a liquid collection that results in abscess <4 cm. A contrast-enhanced CT in the axial plane **(B)** shows the same findings as US. A follow-up colonic endoscopy confirmed the presence of a single diverticulum at that location.

**Figure 6:**
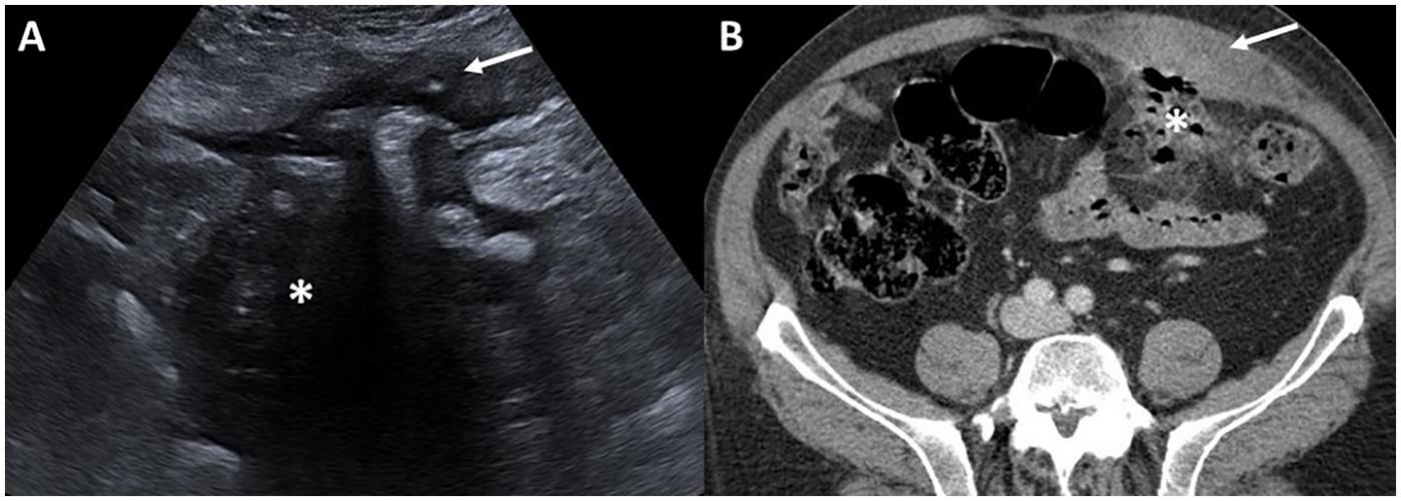
Acute left colonic diverticulitis stage 2 in a patient with previous episodes of diverticulitis. At the time of exploration, a US image obtained with a convex transducer **(A)** shows a locally liquid collection (*) >4 cm close to the colonic wall that seems extend to the abdominal wall. A contrast-enhanced CT in the axial plane **(B)** shows part of the abscess with gas (*) and an inflamed left rectal abdominal muscle (white arrow).

Colour Doppler was used in 31 of the 50 patients with a final diagnosis of ALCD, of whom 26 patients (84%) were assessed by Radiologist A and the remaining 5 (16%) by Radiologists B and C. Seven of the 31 patients (22.6%) had no significant Doppler signal and were diagnosed as stage 0. Only 4 (21%) of the 19 patients without usage of colour Doppler were assessed by Radiologist A. The images of mild and moderate acute diverticulitis are depicted in Figures 3–6.

When comparing the radiological severity of ALCD and laboratory data, no significant difference was found between the level of leukocytosis and stage 0 or >0. A total of 63.3% of stage 0 patients had leukocytosis, while 36.7% had leukocytosis at higher stages. However, the results are different when severity is compared with C-reactive protein (CRP) levels. Significant differences were observed between CRP values and radiological grade of disease. CRP was ≤5.5 mg/dL in 61.8% of patients with stage 0 but only in 25% of patients with stage >0. CRP was >5.5 mg/dL in 38.2% of patients with stage 0 and in 75% of patients with stage >0 (*p* = 0.014). A trend towards a higher grade and the presence of systemic inflammatory response syndrome (SIRS) criteria was also observed.

All patients with ALCD were treated with non-steroidal anti-inflammatory drugs and antibiotics, because at the time of the study, treatment without antibiotics in cases of mild diverticulitis (12, 13) had not yet been routinely established in the hospital.

## Discussion

The present prospective study compared US and CT in the same patients with clinically suspected localised mild-to-moderate ALCD, with both examinations performed within 1 h and before treatment. In this selected cohort, both techniques showed similar diagnostic estimates when compared against a multidisciplinary final diagnosis. These findings support the view that, under the selected conditions of the present study—daytime hours, a university-hospital setting, and examinations performed by dedicated abdominal radiologists—US can be an effective initial imaging technique in selected patients with suspected ALCD. However, because only 63 patients were included and only 50 had a final diagnosis of ALCD, these results should be interpreted cautiously and should not be taken as evidence of equivalence or interchangeability between the two modalities.

An important strength of the study is its pragmatic head-to-head design. Unlike many prior series, both imaging tests were performed in the same patient, by independent radiologists, and before treatment initiation. This design reduces temporal and treatment-related confounding and provides a clinically relevant comparison between modalities. In addition, the final diagnosis was not based on imaging alone but on multidisciplinary consensus integrating clinical course, response to treatment, and follow-up information. Because both index tests contributed to the adjudicated final diagnosis, the reference standard was not fully independent, introducing incorporation bias that may have inflated the apparent diagnostic performance of both modalities.

The results are clinically relevant because the incidence of acute colonic diverticulitis has increased over recent decades (14, 15), while the preferred imaging modality remains debated. A greater use of US could reduce radiation exposure, intravenous contrast administration, and costs.

The study also provides relevant information on severity assessment. Agreement between US and CT for differentiating stage 0 from stage >0 ALCD was moderate overall. Most discordant cases involved underestimation of severity by US within the lower severity spectrum, and the few misclassified complicated cases were predominantly limited-stage disease. This suggests that US may be particularly useful as an initial imaging tool in patients with clinically suspected mild-to-moderate localised ALCD, while CT remains important when complications are suspected, when the ultrasound result is inconclusive, or when management planning requires a more complete staging.

A key finding of the study is the operator dependence of intestinal ultrasonography. Agreement between US and CT was higher when US was performed by the radiologist with greater experience in emergency abdominal ultrasound, and the use of colour Doppler also varied according to the operator. This observation has direct implications for reproducibility and generalisability. The good performance of US shown here should not be considered an intrinsic property of the technique alone, but rather a result achievable in a setting with trained abdominal radiologists and access to appropriate equipment. The present study was not designed to define a learning curve or a minimum number of examinations required to achieve competency; however, it strongly suggests that structured training and protocol standardisation are essential if similar results are to be reproduced by radiologists, gastroenterologists, or emergency physicians in other settings.

A broader real-world perspective should also be considered when interpreting these findings. Ultrasound-based diagnostic pathways for acute diverticulitis may have relevance beyond specialised radiology units, and recent reports have described the use of point-of-care ultrasound in other clinical environments (16). However, the results should not be directly extrapolated to non-radiologist settings. Rather, one of the main messages of the present study is that the diagnostic performance of ultrasonography in suspected ALCD appears to be closely linked to operator expertise, specific training in abdominal ultrasound, and a structured radiological workflow. The favourable results observed here were obtained under controlled conditions by experienced abdominal radiologists, and this context should be taken into account when considering the applicability of our findings to other emergency care settings.

Evidence comparing US and CT remains limited. Systematic reviews and a meta-analysis have reported similar sensitivity and specificity for both techniques (17, 18), supporting either as an initial test, although CT may better identify alternative diagnoses. Guideline recommendations vary geographically: US is widely used in Europe and endorsed as first-line imaging (19–23), whereas North American guidance generally recommends CT (24–26); a recent US update again favoured CT as the only recommended diagnostic test (3).

Few comparative studies have performed both examinations in the same patients. A prospective study of 64 patients reported a similar rate of accuracy (84%) for US and CT (27). The present results are consistent with higher performance metrics, plausibly reflecting technical advances in US. In a larger cohort, US followed by CT was used to classify complicated vs. uncomplicated diverticulitis in accordance with World Society for Emergency Surgery (WSES) guidance (20), yielding a sensitivity of 84% and specificity of 95.8% for complicated disease; most misclassifications involved stage Ia on CT (27). Similarly, severity was underestimated in a small proportion of patients in the present study, and these patients were subsequently classified as stage Ia on CT; none required surgery. One stage IV case was understaged as stage II on US. Notably, in most patients, the initial US assessment would have been sufficient, potentially avoiding CT in patients initially considered to have mild-to-moderate ALCD. Agreement for a simplified classification (stage 0 vs. >0) improved when US was performed by the most experienced radiologist.

An increased prevalence of diverticulitis in younger male patients has been reported (14), and age- and sex-related differences were also observed in the present cohort, supporting a preference for US to limit radiation exposure in younger patients. CRP was elevated, consistent with prior work (9), and higher CRP levels correlated with greater radiological severity, as previously described (28). However, this observed association between CRP and radiological severity should be interpreted as exploratory and hypothesis-generating, given the modest sample size of the study and the limited number of higher-stage cases.

This study has limitations. The sample size was modest, with few high-severity or complicated cases, limiting some comparisons and generalisability. The wide confidence intervals, particularly for specificity, indicate that the absence of a statistically significant difference should not be interpreted as proof of equivalence. In addition, this study was conducted under favourable daytime conditions with subspecialised radiologists, which may not reflect all emergency settings. Variability in colour Doppler use may also have influenced US performance. In the present study, body mass index was not an exclusion criterion, and no patient was excluded because of obesity or bowel-gas artefact. Nevertheless, the study was not powered to evaluate the impact of obesity on image quality or diagnostic accuracy, and increased abdominal wall thickness or deeper target location may reduce ultrasound performance, particularly with high-frequency linear probes. Another limitation is that, although the radiologists were blinded to the counterpart imaging results, they were aware of the clinical suspicion of ALCD at the time of image interpretation, which may have introduced diagnostic expectation bias. A strength of this study is that all patients underwent both examinations, which were performed and interpreted by a small group of radiologists within a blinded, standardised workflow, thereby enhancing internal validity.

From a clinical standpoint, the present data support a cautious stepwise imaging strategy. In haemodynamically stable patients with localised left lower quadrant pain, no diffuse peritonitis, and no signs of sepsis or organ dysfunction, US performed by a trained operator may be considered as the initial imaging test. If US demonstrates uncomplicated or mildly complicated localised diverticulitis in a manner concordant with the clinical presentation, CT may be avoided initially. By contrast, CT should be performed in patients with inconclusive or negative US despite persistent clinical suspicion, poor acoustic window, marked obesity or bowel gas, suspicion of alternative diagnoses, or sonographic findings suggestive of more advanced complications. In unstable patients or in those with non-localised abdominal pain, sepsis, or organ dysfunction, CT should remain the preferred first-line modality. This approach is in line with recommendations issued by European scientific societies, including EFSUMB (24).

The proposed imaging strategy is consistent with the study cohort, the institutional flowchart already included in the manuscript, and the observed operator dependence of US. Although broader implementation might reduce unnecessary CT utilisation and radiation exposure, particularly in younger patients, these data principally support use in centres with dedicated expertise in abdominal ultrasonography rather than unrestricted generalisation to all emergency settings. These findings also support ensuring access to radiologists trained in abdominal US and intestinal Doppler techniques beyond routine daytime hours.

## Conclusion

In this prospective cohort of selected, clinically stable patients with suspected localised mild-to-moderate ALCD, ultrasonography performed by experienced abdominal radiologists showed similar diagnostic estimates to CT and correctly identified most final ALCD cases. However, the limited sample size, the small number of advanced complications, the operator-dependent nature of intestinal ultrasonography, and the non-independent reference standard require cautious interpretation. These findings support the use of US as a possible initial imaging approach in specialised centres and selected patients, but they do not establish equivalence or interchangeability with CT (29).

## Data Availability

The raw data supporting the conclusions of this article will be made available by the authors, without undue reservation.
