# Supplementary material for: Ultrasonography for suspected mild-to-moderate acute left colonic diverticulitis: a prospective head-to-head study with computed tomography
Source: Front Radiol. 2026 May 28;6:1829813. doi: 10.3389/fradi.2026.1829813 (PMC13253527; doi:10.3389/fradi.2026.1829813)
Supplement: Supplementary file 1 [file Table1.docx]

**Supplementary Table 1. Patients whose final diagnosis was other than ALCD (n = 13).**

| **Final diagnosis** | **n** | **US diagnosis** | **CT diagnosis** |
| --- | --- | --- | --- |
| Acute gastroenteritis | 1 | ALCD | ALCD |
| Nonspecific abdominal pain | 1 | Diverticulosis without diverticulitis | Diverticulosis without diverticulitis |
| Ureterolithiasis | 2 | Ureterolithiasis | Ureterolithiasis |
| Right diverticulitis | 1 | No ALCD | Right diverticulitis |
| Transverse colonic diverticulitis | 1 | Transverse colonic diverticulitis | Transverse colonic diverticulitis |
| Acute gastroenteritis | 1 | No US pathological findings | No CT pathological findings |
| Pelvic inflammatory disease | 1 | Pelvic inflammatory disease | Pelvic inflammatory disease |
| Non-complicated appendicitis | 1 | Inconclusive | Non-complicated appendicitis |
| Sigmoiditis of unknown origin | 1 | Sigmoiditis without diverticula | Sigmoiditis without diverticula |
| Urinary tract infection | 1 | No US pathological findings | No CT pathological findings |
| Acute gastroenteritis | 1 | No US pathological findings | No US pathological findings |
| Epiploic appendagitis | 1 | Epiploic appendagitis | Epiploic appendagitis |
